# Deletion of Lkb1 in adult mice results in body weight reduction and lethality

**DOI:** 10.1038/srep36561

**Published:** 2016-11-08

**Authors:** Tizhong Shan, Yan Xiong, Shihuan Kuang

**Affiliations:** 1 College of Animal Sciences, Zhejiang University, Hangzhou, 310058, China; 2 Department of Animal Sciences, Purdue University, West Lafayette, IN 47907, USA; 3 Purdue University Center for Cancer Research, West Lafayette, IN 47907, USA

## Abstract

Liver kinase B1 (Lkb1) plays crucial roles in development, metabolism and survival. As constitutive knockout of Lkb1 in mice leads to embryonic lethality, whether Lkb1 is required for the growth and survival of adult mice is unclear. Here we address this question using a tamoxifen-inducible Lkb1 knockout (KO) mouse model: *Rosa26-Cre*^*ER*^: *Lkb1*^*flox/flox*^ (abbreviated as *Rosa-Lkb1*). The *Rosa-Lkb1* mice exhibited body weight reduction and died within 6 weeks after tamoxifen induction. The body weight reduction was due to reduced weight of various tissues but the brown and white adipose tissues underwent much more pronounced weight reduction relative to the overall body weight reduction. Accordingly, the *Rosa-Lkb1* mice had increased blood glucose levels and were intolerant to glucose challenge. Expression levels of adipogenic and lipogenic genes in adipose tissues were also dramatically reduced by Lkb1 deletion. Additionally, Lkb1 deletion reduced lipid deposition and increased expression of mitochondrial (*Pgc1a, Cox5b* and *Cox7a*) and hepatic gluconeogenesis related genes (*Pepck*) in liver. Finally, the *Rosa-Lkb1* mice had much reduced oxygen consumption, carbon dioxide production, and energy expenditure. These results demonstrate that Lkb1 plays an important role in maintaining body weight, liver and adipose tissue function, blood glucose homeostasis and survival in adult mice.

Liver kinase B1 (Lkb1), also known as serine/threonine protein kinase 11 (Stk11), plays crucial roles in embryo and tissue development, energy metabolism and survival^1^,^2^,^3^,^4^,^5^. Lkb1 acts as a central metabolic sensor of glucose and lipid metabolism by phosphorylating and regulating the AMP-activated protein kinase (AMPK) signaling pathway^5^,^6^. In addition, Lkb1 is a ‘master’ protein kinase that functions upstream of 13 other kinases of the AMPK subfamily^6^.

Previous studies have shown that constitutive knockout of Lkb1 causes severe developmental defects and the KO embryos die at embryonic day 8.5–11^1^. Cardiac-specific deletion of Lkb1 causes early defects in atrial channel expression, atrial fibrillation, hypertrophy and heart dysfunction^7^,^8^,^9^. Liver-specific deletion of Lkb1 in mice results in defective canaliculi and bile duct formation, hyperglycemia, body weight reduction and death^2^,^10^,^11^,^12^. Skeletal muscle-specific deletion of Lkb1 regulates muscle development, lipid oxidation, glucose homeostasis, and insulin sensitivity^13^,^14^,^15^,^16^. In addition, tissue-specific deletions of Lkb1 in adipose tissue^17^,^18^, pancreas^19^,^20^,^21^ and brain^22^,^23^ further demonstrate that Lkb1 plays important roles in these tissues. Moreover, loss of Lkb1 regulates haematopoietic stem cells’ metabolism and survival^3^,^4^,^5^, muscle stem cells’ proliferation and differentiation^16^,^24^, and pancreatic β cells’ polarity^20^. However, due to the embryonic lethality of global knockout of Lkb1 in mice^1^, the role of Lkb1 in tissue homeostasis and survival of adult mice is unclear.

Therefore, in the current study, we used the *Cre-LoxP* system to generate a tamoxifen (TMX)-inducible whole-body Lkb1 knockout mouse model (*Rosa26-Cre*^*ER*^*:Lkb1*^*flox/flox*^, abbreviated as *Rosa-Lkb1*) by crossing *Lkb1*^*flox/flox*^ mice with *Rosa26-Cre*^*ER*^ mice. We found several metabolic consequences in the *Rosa-Lkb1* mice that eventually lead to lethality within 6 weeks after TMX induced deletion of Lkb1 in adult mice.

## Results

### Generation of TMX-inducible whole-body Lkb1 knockout mice

**Figure 1:**
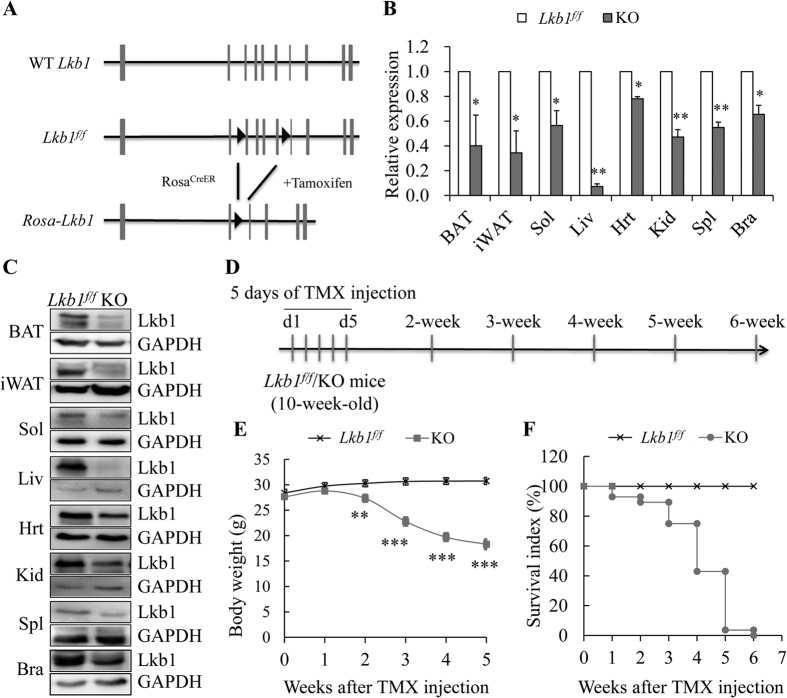
Global deletion of Lkb1 leads to body weight reduction and lethality. (**A**) Targeting strategy. (**B**,**C**) Relative mRNA (**B**) and protein (**C**) levels of *Lkb1* in different tissues (n = 4). (**D**) TMX induction strategy. (**E**,**F**) Body weight (**E**, n = 7–18 ) and survival index (**F**, n = 24–28) of *Lkb1*^*f/f*^ and KO mice. Error bars: S.E.M.; *means *P* < 0.05, **means *P* < 0.01, and ***means *P* < 0.001 unless otherwise indicated in figure legends.

To directly investigate the effects of global Lkb1 knockout in adult mice, we used the *Cre-LoxP* recombination system. We generated TMX-inducible whole-body Lkb1 knockout mice *Rosa-Lkb1* by crossing the *Lkb1*^*flox/flox*^ mice with the *Rosa26-Cre*^*ER*^ mice (Fig. 1A). In adult *Rosa-Lkb1* mice, TMX injection activates *Cre* in all cells due to the ubiquitous expression of the *Rosa*26 gene. Indeed, real-time PCR analysis showed a dramatic reduction of Lkb1 in brown adipose tissue (BAT), inguinal white adipose tissue (iWAT), soleus muscle (Sol), heart (Hrt), kidney (Kid), spleen (Spl), and brain (Bra) (Fig. 1B). Consistently, western blot results confirmed the efficient reduction of Lkb1 in various tissues (Fig. 1C). Notably, the Lkb1 gene was robustly deleted in adult liver (Liv) (Fig. 1B,C). These results demonstrate the utility of the *Rosa-Lkb1* mouse model for temporally controlled deletion of the Lkb1 gene after TMX induction.

### Global deletion of Lkb1 in adult mice causes body weight reduction and lethality

To examine the role of Lkb1 in adult mice, TMX (300 μl per mouse, 10 mg/ml) was injected intraperitoneally (I. P.) to 10-wk-old mice once a day for 5 consecutive days (Fig. 1D). After TMX injection, we examined the body weight changes of the mice (Fig. 1D). Although the body weights of the *Lkb1*^*flox/flox*^ and *Rosa-Lkb1* mice were similar before TMX injection, the body weight of the *Rosa-Lkb1* mice began to decrease from 2 weeks after TMX induction (Fig. 1E). At 5 weeks after TMX injection, the body weight of the *Rosa-Lkb1* mice was only 18.30 ± 0.34 g, about 40% lighter than that of the control *Lkb1*^*flox/flox*^ littermates (30.75 ± 0.74 g) (Fig. 1E). Notably, we also found that the *Rosa-Lkb1* mice began to die from 1 week after TMX injection (Fig. 1F). At 4 weeks, more than 57% KO mice died, and no KO mice survived beyond 6 weeks after TMX injection (Fig. 1F). In contrast, all *Lkb1*^*flox/flox*^ mice survived after TMX treatment (Fig. 1F). These results indicate that Lkb1 plays an important role in maintaining body weight and survival of adult mice.

### Global deletion of Lkb1 in adult mice decreases the ratio of WAT to body weight

**Figure 2:**
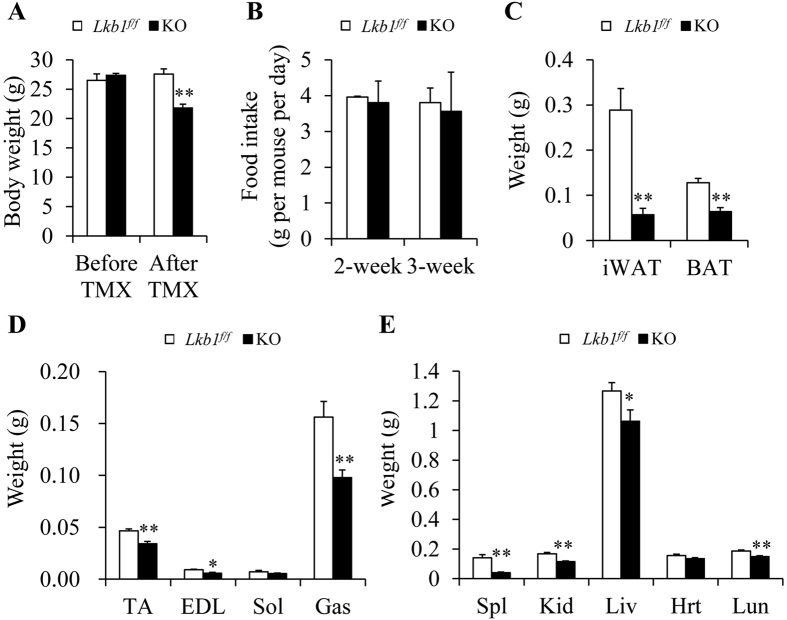
Lkb1 deletion reduces body weight and tissue mass. (**A**) Body weight of *Lkb1*^*f/f*^ (n = 8) and KO (n = 12) mice at 3 weeks after TMX induction. (**B**) Food intakes of *Lkb1*^*f/f*^ and KO mice at 2- and 3-week after TMX injection (n = 6). (**C–E**) The weight of fat (**C**), muscle (**D**) and organs (**E**) from *Lkb1*^*f/f*^ (n = 6) and KO (n = 9) mice.

To examine how global deletion of Lkb1 decreased the body weight of adult mice, we investigated the weight of various tissues at 3 weeks after the start of TMX administration. At that time, the body weights of the KO mice were also less than that of the *Lkb1*^*flox/flox*^ mice, though the body weights were similar before TMX treatment (Fig. 2A). Similar food intake was found between *Lkb1*^*flox/flox*^ and KO mice at 2- and 3-weeks after TMX injection (Fig. 2B). Upon examination of various tissues, we found that global deletion of Lkb1 significantly decreased the weights of iWAT, BAT, TA, EDL and Gas muscles, as well as various organs including spleen, kidney, liver and lung (Fig. 2C–E). When normalized to the body weight, only the relative weights of iWAT, BAT and spleen were lower in KO compared to control mice, but the relative weights of muscle and other organs including liver, heart and lung to body weight were unchanged or even higher in KO mice than that of *Lkb1*^*flox/flox*^ mice (Supplemental Fig. 1A–C). As the spleen only accounts for a very small fraction of body weight, these results indicate that global deletion of Lkb1 in adult mice leads to decreases in body weight mainly due to the reduction of adipose tissues.

**Figure 3:**
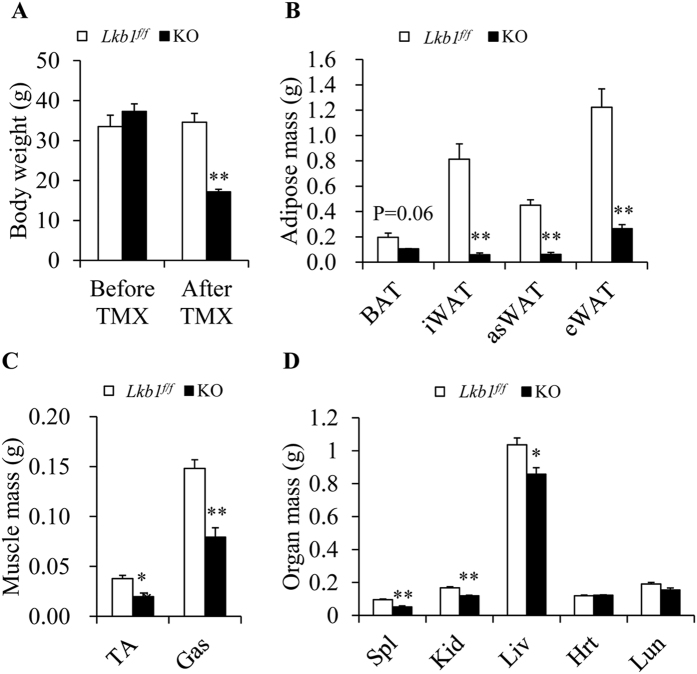
Global deletion of Lkb1 decreases body weight and fat mass of the HFD-fed KO mice. (**A**) Body weight of HFD-induced mice before or after TMX induction. (**B–D**) The weight of fat (**B**), muscle (**C**) and organs (**D**) from HFD-induced mice after TMX induction (n = 3–4).

To further examine the effects of global deletion of Lkb1 on fat mass and to determine if the lethality in KO mice is due to reduction of fat mass, we used high-fat diet (HFD) to promote fat deposition prior to induced-deletion of Lkb1. After 10 weeks of HFD treatment, both *Lkb1*^*flox/flox*^ and *Rosa-Lkb1* mice were obese and then treated with TMX to delete Lkb1. We found that the HFD-fed mice still died within 6 weeks after TMX induced deletion of Lkb1 (data not shown). Though the body weights of the *Lkb1*^*flox/flox*^ and *Rasa-Lkb1* mice were similar before TMX, the body weights of KO mice (17.16 ± 0.62 g) were ~50% lighter than that of *Lkb1*^*flox/flox*^ mice (34.6 ± 2.18 g) at 4 weeks after TMX injection (Fig. 3A). Notably, the WAT mass of the KO mice was dramatically decreased (Fig. 3B). Furthermore, we found that the weights of the other tissues including TA, Gas, Kid, Spl, and Liv were also significantly decreased (Fig. 3C,D). However, the ratios of iWAT, asWAT and eWAT to body weight were lower, but the ratios of kidney, liver, heart and lung to body weight were unchanged or even higher in KO mice than that of *Lkb1*^*flox/flox*^ mice (Supplemental Fig. 2A–C). Therefore, HFD fails to prevent from body weight loss and lethality in the Lkb1 KO adult mice.

**Figure 4:**
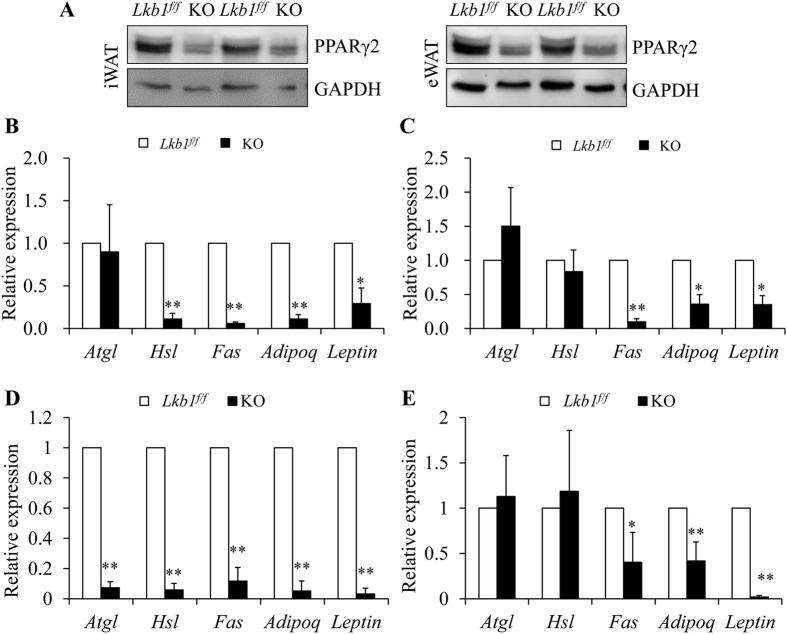
Global deletion of Lkb1 alters gene expression in WAT. (**A**) Protein levels of PPARγ2 in WAT from HFD-fed mice after TMX induction. (**B–E**) Relative expression of fat deposition related genes in WAT from HFD- (**B**, iWAT; **C**, eWAT) or normal diet-fed (**D**, iWAT; **E**, eWAT) mice after TMX induction (n = 3–4).

At the gene expression level, the relative protein levels of the adipocyte differentiation related PPARγ2 were decreased in iWAT and eWAT of the KO mice compared to that of the control mice (Fig. 4A). Real time PCR results also showed that the mRNA levels of the fat deposition related gene *Fas* and the mature adipocytes markers *Adipoq* and *Leptin* were significantly decreased in adipose tissues of HFD-fed (Fig. 4B,C) or from normal fat diet (NFD)-fed (Fig. 4D,E) *Lkb1*^*flox/flox*^ and KO mice after TMX induction. Taken together, these results showed that reduction of adipose tissues (both brown and white) is correlated to changes in the expression of adipogenic genes in the Lkb1 KO adult mice.

### Deletion of Lkb1 affects glucose metabolism

**Figure 5:**
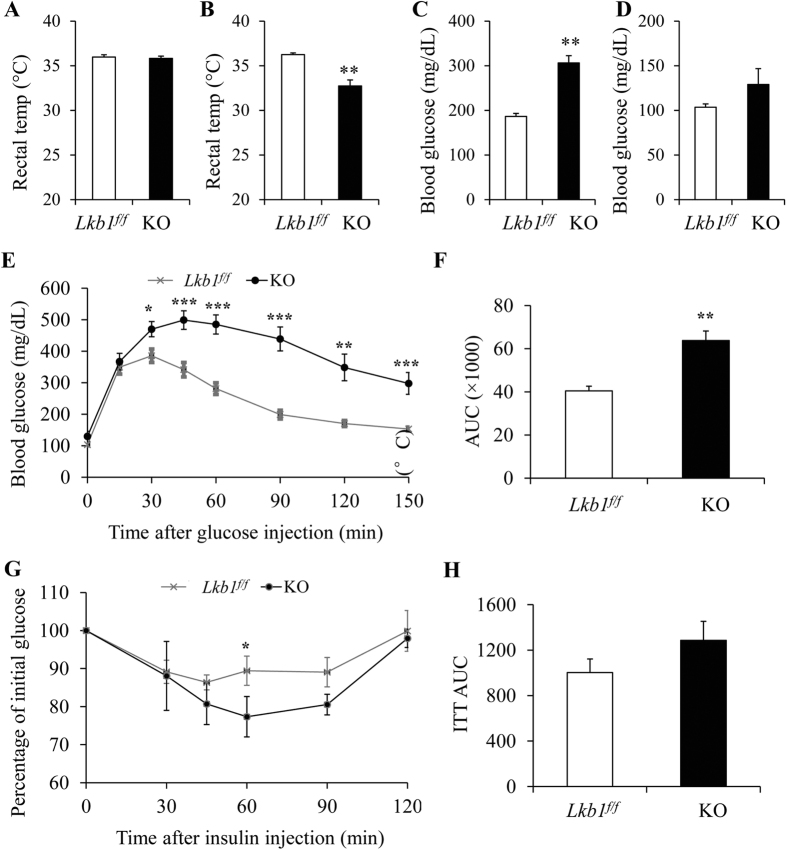
Global deletion of Lkb1 results in glucose intolerance. (**A**,**B**) Rectal temperature of *Lkb1*^*f/f*^ and KO mice before (**A**, n = 6) or after (**B**, n = 14) TMX induction. (**C**,**D**) Blood glucose before (**C**) or after fasting16 h (**D**) (n = 8–10). (**E–H**) Blood glucose and the area under curve (AUC) during GTT (**E**,**F**; n = 8–10) and ITT (**G**,**H**; n = 5–6).

**Figure 6:**
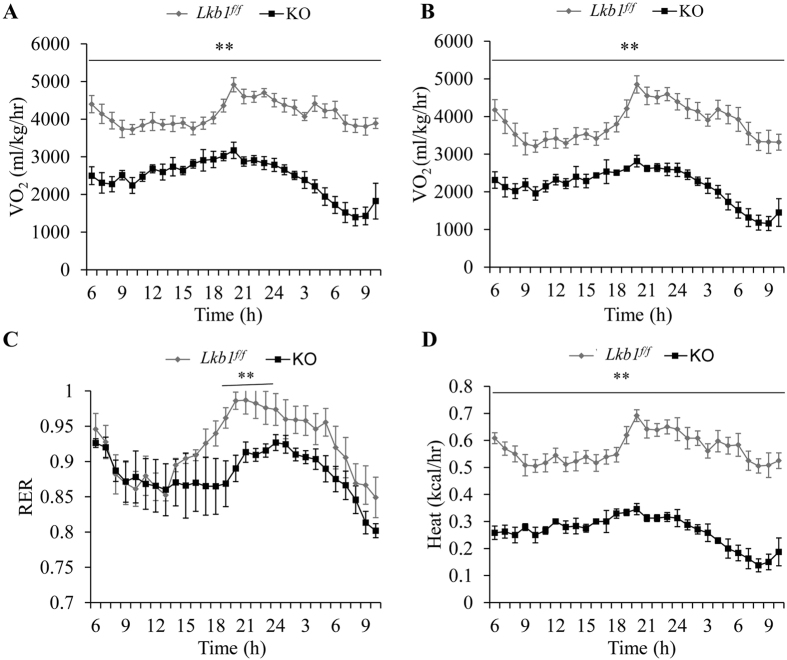
Lkb1 deletion decreases energy expenditure. **(A–D)** The oxygen consumption (VO_2_, **A**), carbon dioxide production (VCO_2_, **B**), respiratory exchange ratio (RER, **C**) and heat production (**D**) of *Lkb1*^*f/f*^ (n = 6) and KO (n = 4) mice after TMX injection.

As Lkb1 KO in adult mice affects both BAT and WAT mass, we next determined if the changes in fat mass are associated with alterations in thermoregulation and energy metabolism. We first measured the rectal temperatures, as an indication of heat production by BAT, of the *Lkb1*^*flox/flox*^ and KO mice before and after TMX injection. We found the rectal temperature of the KO mice was lower compared to the control mice at 3 weeks after TMX injection, though similar rectal temperature was found between *Lkb1*^*flox/flox*^ and KO mice before TMX (Fig. 5A,B). The blood glucose levels of the KO mice were higher than that of *Lkb1*^*flox/flox*^ mice at 3 weeks after TMX injection (Fig. 5C,D). Furthermore, compared to the *Lkb1*^*flox/flox*^ littermates, *Rosa-Lkb1* mice had higher blood glucose levels after intraperitoneal (IP) injection of glucose (Fig. 5E) and lower blood glucose levels at 60 minutes after insulin injection (Fig. 5G). The area under the curve (AUC) of the GTT, but not the AUC of the ITT, showed significant difference between *Lkb1*^*f/f*^ and KO mice (Fig. 5F,H). In addition, the *Rosa-Lkb1* mice had lower rates of oxygen consumption and carbon dioxide production and expended less energy compared to the *Lkb1*^*flox/flox*^ mice (Fig. 6). Taken together, these results suggest that global deletion of Lkb1 in adult mice leads to glucose intolerance and decreases energy metabolism.

### Deletion of Lkb1 affects liver function and glucose homeostasis

We found that the expression of Lkb1 was nearly abolished in liver tissues of the *Rosa-Lkb1* mice (Fig. 1). To examine the effects of Lkb1 deletion on liver tissues, we injected Evans Blue (EB) dye to *Lkb1*^*flox/flox*^ and KO mice at 3 weeks after TMX injection. We found that EB uptake was more pronounced in the liver tissues of KO mice compared to control mice (Supplemental Fig. 3A), indicating Lkb1 deletion may induce liver degeneration. Staining results also showed that deletion of Lkb1 reduced lipid deposition in liver (Supplemental Fig. 3B). Indeed, quantification results showed that deletion of Lkb1 dramatically decreased triglyceride content in the liver from the KO mice compared to that of *Lkb1*^*flox/flox*^ mice (Supplemental Fig. 3C). Moreover, we checked the gene expression and found that the mitochondrial related genes including *Pgc1a, Cox5b* and *Cox7a* were significantly upregulated in the KO livers (Supplemental Fig. 3D). Deletion of Lkb1 also increased the expression of *Pepck* gene, a rate-determining enzyme in hepatic gluconeogenesis, in the liver (Supplemental Fig. 3D), indicating defective liver glucose homeostasis. The expression of *Fas* and *Srebp1c*, however, was similar between *Lkb1*^*flox/flox*^ and KO mice (Supplemental Fig. 3D). These results suggested that deletion of Lkb1 affects liver function and liver glucose homeostasis.

## Discussion

In this study, we generated a TMX-inducible whole-body Lkb1 knockout mice model and examined the effects of global Lkb1 ablation on adult mice. We found that global deletion of Lkb1 in adult mice reduces body weight and leads to lethality within 6 weeks after TMX induction. Moreover, the absolute and relative masses of adipose tissues are dramatically decreased in the KO mice compared to the *Lkb1*^*flox/flox*^ mice. Furthermore, global deletion of Lkb1 in adult mice affects the glucose metabolism, liver function and liver glucose homeostasis. These results indicate that Lkb1 is necessary for maintaining adult mice body weight, liver and adipose tissue function, blood glucose homeostasis, and animal survival.

Lkb1 plays a major role in regulating of metabolism and controlling physiological functions of many tissues^2^,^9^,^16^,^17^. Here we found that TMX-inducible whole-body deletion of Lkb1 in adult mice reduces fat mass and body weight under chow diet or HFD diet. Likewise, several tissue-specific Lkb1 deletion mice models also exhibited a similar phenotype in body weight reduction. For example, *FABP4-Cre* induced adipose-specific Lkb1 knockout mice (*FABP4-Lkb1*) exhibited a reduced amount of WAT and postnatal growth retardation^17^. However, *Adiponectin-Cre* mediated adipocyte-specific deletion of Lkb1 resulted in normal WAT mass and body weight but enhanced BAT mass and induced browning of WAT^18^. As *Fabp4-Cre* is expressed in mature adipocytes, preadipocytes, and non-adipose tissues including endothelial cells^25^,^26^, the body weight reduction and premature death of the *Fabp4-Lkb1* KO mice may be due to the role of Lkb1 in preadipocytes or non-adipose effects^25^,^26^. Muscle-specific deletion of Lkb1 caused skeletal muscle dysfunction and reduced body weight in the old mutant mice^27^. In our previous study, we deleted Lkb1 in muscle progenitors and muscle tissues using *MyoD-Cre* and found the *MyoD-Lkb1* mice were born smaller and grew more slowly than their WT littermates^16^. But the lower body weight of the *MyoD-Lkb1* mice was mainly due to the reduction of muscle mass. Mice lacking Lkb1 in the liver were smaller than WT mice and rapidly began to lose weight from around 15 days of age^11^. *RIP2-Cre* mediated deletion of Lkb1 in pancreatic β-cell decreased body weight and daily food intake, improved glycemia but did not alter insulin sensitivity^21^. However, other studies showed that deletion of Lkb1 in all pancreatic lineage cells^28^,^29^ did not affect body weights. Here we also found that the heart of KO mice appeared larger than that of the *Lkb1*^*flox/flox*^ mice. Consistently, cardiac-specific deletion of Lkb1 resulted in the hypertrophy and dysfunction of heart^9^ but did not affect the body weight^7^,^8^. Taken together, the reduction of body weight in the TMX-inducible Lkb1 knockout mice may be mainly driven by the Lkb1 deficiency in liver or endothelial cells, and the reduction of fat mass may be a secondary effect of altered systemic energy metabolism.

We found that TMX-inducible whole-body deletion of Lkb1 in adult mice causes severe hyperglycemia and glucose intolerance. Likewise, higher fasting blood glucose levels were found in liver-specific Lkb1 deletion mice^2^. Mice lacking hepatic Lkb1 had glucose intolerance after injection of glucose, and normal reduction in blood glucose in response to insulin injection^2^. Hepatocyte-specific deletion of Lkb1 in adult mice demonstrated its critical role in control of hepatic glucose homeostasis^2^,^10^. Liver-specific ablation of Lkb1 causes increased glucose production in hepatocytes *in vitro* and hyperglycaemia in fasting mice *in vivo*^30^. In contrast, deletion of Lkb1 in all pancreatic lineages show marked improvements in glucose tolerance but did not affect body weight^29^. The blood glucose levels of *FABP4-Lkb1* mice were markedly lower than those of WT mice^17^. However, the *Adipoq-Lkb1* mice have improved glucose tolerance and insulin sensitivity^18^. In addition, skeletal muscle-specific deletion of Lkb1 improves glucose tolerance and affects muscle glucose uptake^13^,^14^,^15^. Taken together, our current and previous data suggest that the expression of Lkb1 in liver is beneficial for maintaining normal blood glucose concentrations.

We found that TMX-inducible whole-body Lkb1 knockout adult mice died within 6 weeks after TMX induction. It has been reported that Lkb1 global knockout embryos die at E8.5–11 with severe developmental defects, such as defective vasculature, and mesenchymal tissue cell death^1^. Likewise, *Tie1-Cre* or *Tie2-Cre* mediated ablation of Lkb1 in hematopoietic lineages and neurological tissues also results in embryonic lethality^31^,^32^. Recently, Zhang *et al*. reported that the endothelial cell-specific deletion of Lkb1 causes endothelial dysfunction and only about 5.6% of the KO mice survived into adulthood^33^. Deletion of Lkb1 in the haematopoietic system impairs haematopoietic stem cell quiescence, survival and metabolism, which results in rapid ultimately animal death^4^,^5^. The KO mice died within 30 days post completing tamoxifen injection^4^,^5^. Lkb1 plays important roles in the cardiovascular system^1^. Cardiac-specific deletion of Lkb1 using *MHC-Cre* leads to hypertrophy cardiac dysfunction and the KO mice died within 6 months of age^7^,^9^. *FABP4-Lkb1* mice began to die at day 3 after birth and typically died within 3 weeks after birth^17^. However, *Adipoq-Cre* medicated deletion of Lkb1 does not affect the survival of young mice. Moreover, we found that some *MyoD-Lkb1* mice began to die after weaning, and reach up to 45% mortality within 6 months^16^. Consistent with our findings, liver-specific knockout mice exhibited severe metabolic defects, substantial weight loss, and died within 6 weeks^11^,^12^. In addition, Lkb1 is required for hepatic canalicular membrane integrity and liver function^11^,^12^. Liver-specific deletion of Lkb1 leads to defective bile duct and canalicular network formation^11^,^12^. More recently, Porat-Shliom *et al*. directly demonstrated that Lkb1 plays a key role in maintaining the functional tight junction and paracellular permeability^34^, which may explain why there were more EB uptake in liver tissues of *Rosa-Lkb1* mice compared to control mice. These findings combined with our results indicated that the lethality of *Rosa-Lkb1* mice maybe mainly due to the ablation of Lkb1 in liver or haematopoietic stem cells. However, more studies are needed to examine the exact reason for the lethality of the *Rosa-Lkb1* adult mice.

In conclusion, we generated a TMX-inducible whole-body Lkb1 knockout mice model and used this model to show that global deletion of Lkb1 reduces body weight, leads to hyperglycaemia and glucose intolerance, accompanied by morphological, functional and gene expression changes in the liver. Our results demonstrate that Lkb1 plays important roles in maintaining body weight, liver and adipose tissue function, blood glucose homeostasis and survival in adult mice.

## Material and Methods

### Animals

The *Rosa26-Cre*^*ER*^ (stock number: 008463) and *Lkb1*^*flox/flox*^ (stock number: 014143) mouse strains were bought from Jackson Laboratory (Bar Harbor, ME). Mice were housed in the animal facility with free access to water and diet. All procedures involving mice were performed in accordance with Purdue University Animal Care and Use Committee. Additionally, all experimental protocols were approved Purdue University Animal Care and Use Committee.

### Indirect calorimetry study

The oxygen consumption (VO_2_), carbon dioxide production (VCO_2_), respiratory exchange ratios (RER) and heat production were measured by using indirect calorimetry system (Oxymax, Columbus Instruments) as previously described^35^.

### Glucose tolerance tests (GTT) and insulin tolerance tests (ITT)

At 3 weeks after TMX induction, the glucose tolerance tests (GTT) and insulin tolerance tests (ITT) were measured as previously described^35^. Briefly, for GTT, mice were fasted for 16 h before intraperitoneal injection of 100 mg/ml D-glucose (2 g/kg body weight). For ITT, mice were given intraperitoneal injection of human insulin (Santa Cruz) (0.75 U/kg body weight) after fasting for 4 h. After injection, the tail blood glucose concentrations were monitored.

### Hematoxylin-eosin (H&E) and Oil Red O staining

Frozen livers were cut into 10 μm thick cross sections using a Leica CM1850 cryostat for H&E and Oil Red O (ORO) staining. For HE staining, liver sections were stained as previously described^16^. For ORO staining, liver sections were stained using Oil red O work solutions containing 6 ml stock solution (5 g/L in isopropanol) and 4 mL ddH_2_O for 30 min.

### Total RNA extraction, cDNA synthesis and real-time PCR

Total RNA was extracted using Trizol Reagent and the concentration of total RNA was measured by Nanodrop 3000 (Thermo Fisher) at 260 nm and 280 nm. Then 5 μg of total RNA were reverse transcribed using MMLV reverse transcriptase and real-time PCR was carried out. The 18S rRNA was used as internal control and the relative expression of related genes was analyzed using 2^−∆∆CT^ method.

### Western blot analysis

Total protein was extracted using RIPA buffer^16^. Total proteins were separated by SDS-PAGE, transferred to PVDF membrane (Millipore Corporation) and determined with specific primary antibodies. The PPARγ2, Lkb1, FABP4 and GAPDH antibodies are from Santa Cruz Biotechnology (Santa Cruz). The horseradish peroxidase (HRP)-conjugated secondary antibody (anti-rabbit IgG, 111-035-003 or anti-mouse IgG; 115-035-003, Jackson ImmunoResearch) were diluted 1: 10,000. Immunodetection was performed using enhanced chemiluminescence western blotting substrate (Pierce Biotechnology) and detected with FluorChem R System (ProteinSimple).

### Data Analysis

All experimental data are presented as means ± SEM. Comparisons were made by unpaired two-tailed Student’s t-tests or one-way ANOVA, as appropriate. Effects were considered significant at *P* < 0.05.

## Additional Information

**How to cite this article**: Shan, T. *et al*. Deletion of Lkb1 in adult mice results in body weight reduction and lethality. *Sci. Rep.* **6**, 36561; doi: 10.1038/srep36561 (2016).

**Publisher’s note:** Springer Nature remains neutral with regard to jurisdictional claims in published maps and institutional affiliations.

## Supplementary Material

Supplementary Information
