# Supplementary material for: Deletion of Lkb1 in adult mice results in body weight reduction and lethality
Source: Sci Rep. 2016 Nov 8;6:36561. doi: 10.1038/srep36561 (PMC5099919; doi:10.1038/srep36561)
Supplement: Supplementary Information [file srep36561-s1.pdf]

Deletion of Lkb1 in adult mice results in body weight reduction and lethality

Tizhong Shan[1*], Yan Xiong[2], Shihuan Kuang[2,3*]

[1] College of Animal Sciences, Zhejiang University, Hangzhou, 310058， China

[2] Department of Animal Sciences, Purdue University, West Lafayette, IN 47907, USA

[3] Purdue University Center for Cancer Research, West Lafayette, IN 47907, USA

***Correspondence:**

Tizhong Shan, Ph.D., Professor

866 Yuhangtang Road, Hangzhou, China

Phone: +86-0571-88982102

Email: tzshan@zju.edu.cn

Shihuan Kuang, Ph. D., Associate Professor

174B Smith Hall,

901 West State Street,

West Lafayette, IN 47907, USA

Phone: 765-494-8283

Fax: 765-494-6816

Email: skuang@purdue.edu

Running title： Global Lkb1 knockout in adult mice

Supplemental figures

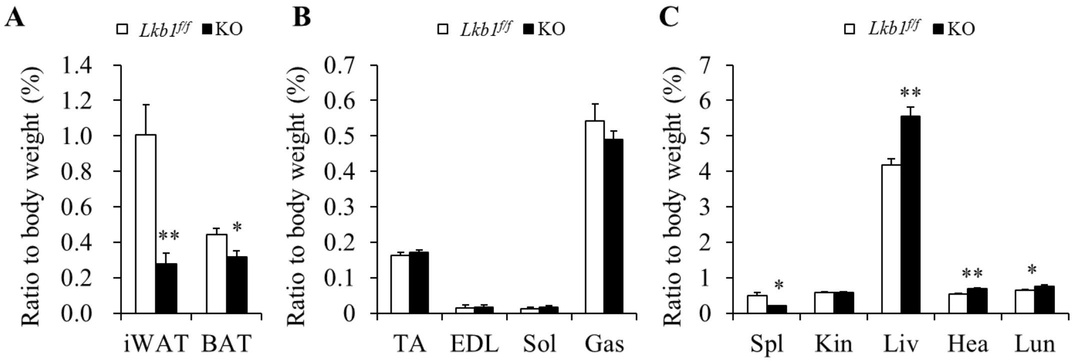

Supplemental Figure 1: The effect of global deletion of Lkb1 on the ratios of different tissues to body weight. (**A**-**C**) The ratios of adipose (**A**), muscle (**B**) and organs (**C**) to body weight of normal diet fed *Lkb1*$^{flox/flox}$ (*Lkb1*$^{f/f}$, n=6) and *Rosa-Lkb1* (KO, n=9) mice at 3 weeks after tamoxifen (TMX) induction. Error bars: S.E.M., * $P < 0.05$, ** $P < 0.01$. BAT, Brown adipose tissue; iWAT, inguinal white adipose tissue; TA, tibialis anterior muscle; EDL, extensor digitorum longus muscle; Sol, soleus muscle; Gas, gastrocnemius muscle; Spl, spleen; Kid, kidney; Liv, liver; Hrt, heart; Lung, Lun.

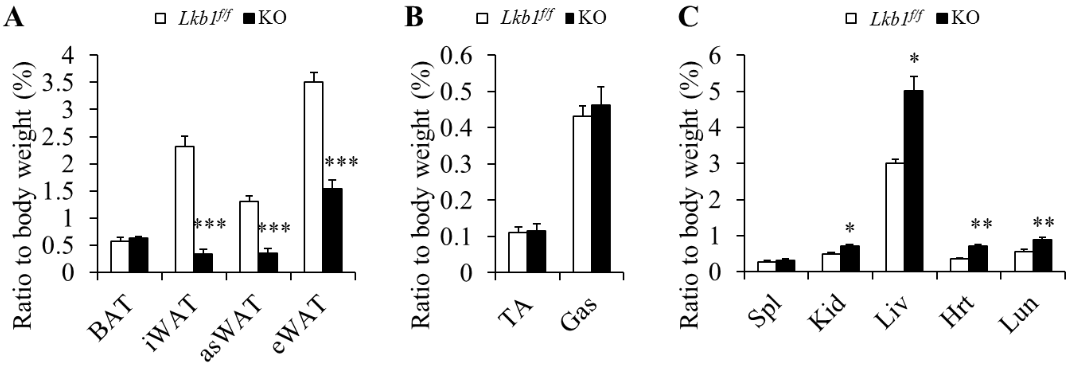

Supplemental Figure 2: The ratios of different tissues to body weight of HFD-induced *Lkb1*$^{flox/flox}$ (*Lkb1*$^{f/f}$) and *Rosa-Lkb1* (KO) mice at 3 weeks after TMX induction. (**A**-**C**) The ratios of adipose (**A**), muscle (**B**) and organs (**C**) to body weight. Error bars: S.E.M., * $P < 0.05$, ** $P < 0.01$. BAT, Brown adipose tissue; iWAT, inguinal white adipose tissue; asWAT, anterior subcutaneous WAT; eWAT, epididymal WAT; TA, tibialis anterior muscle; Gas, gastrocnemius muscle; Spl, spleen; Kid, kidney; Liv, liver; Hrt, heart; Lung, Lun.

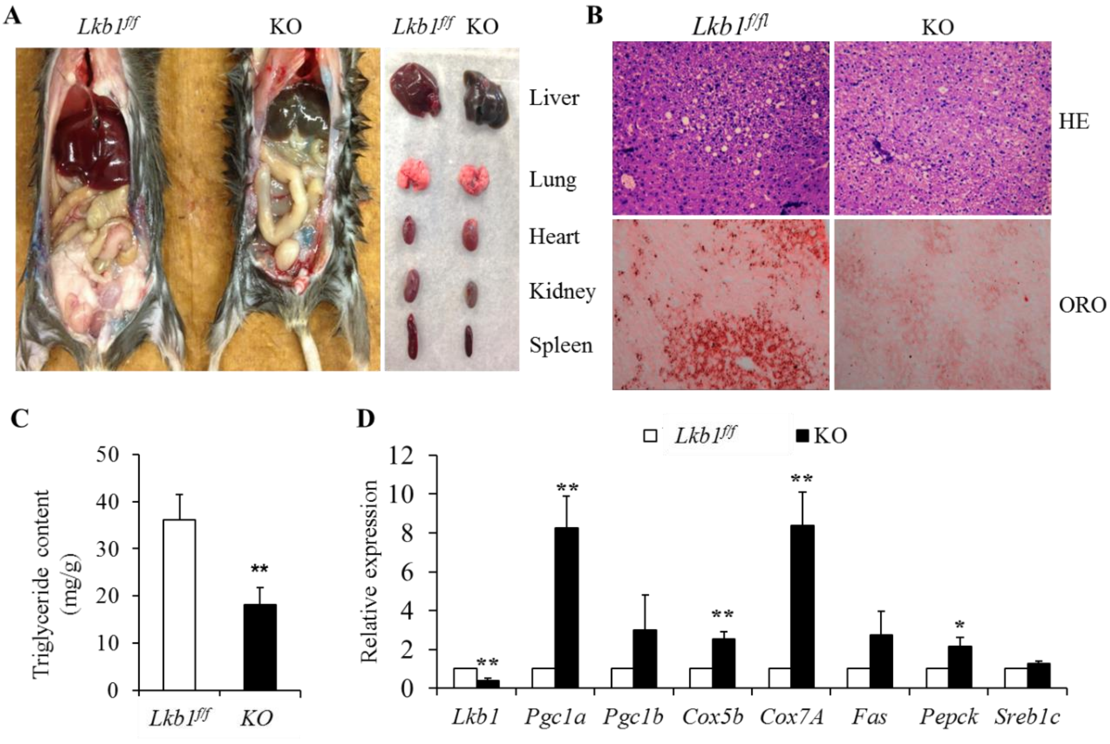

Supplemental Figure 3: Global deletion of Lkb1 affects glycogen and lipid deposition and gene expression in liver. (**A**) Representatives of liver tissues from $Lkb1^{flox/flox}$ ($Lkb1^{f/f}$) and *Rosa-Lkb1* (KO) mice at 3 weeks after tamoxifen (TMX) induction. (**B**) Representatives images of liver sections after HE and oil red O (ORO) staining. (**C**) The triglyceride content in liver tissues (n=4). (**D**) The mRNA levels related genes in liver tissues from $Lkb1^{f/f}$ and KO mice 3 weeks after TMX induction (n=5). Error bars: S.E.M., * $P < 0.05$, ** $P < 0.01$.
